# Office Management

**Published:** 1904-09-15

**Authors:** Henry C. Raymond

**Affiliations:** Detroit, Mich.


### OFFICE MANAGEMENT.

BY HENRY C. RAYMOND, D.D.S., DETROIT, MICH.

Read before the Michigan Dental Association, June 28, 1904.

My treatment of this subject is, of course, largely from the standpoint of a city practitioner, whose ideas differ somewhat from those who practice in villages or small towns. The basis of successful office management, however, should not vary greatly in any practice, and that management that is successful in conducting a city practice will, in a modified degree, be equally successful in conducting practices outside the cities.

Under the head of office management would naturally come the use of an assistant, the disposal or allotment of time, first for operations, second for treatments and consultations, and on the commercial or business side, the bookkeeping and collection of fees.

I will first speak of the assistant, as it is almost impossible to have any kind of management without one in a city practice. In most cases a girl or a young woman is far preferable to a male assistant. I am, of course, not referring to a bench or chair assistant. A girl as a rule is more obliging and willing, is neater, and in nearly every way better adapted for such work. She should be as well educated as possible, and write a good hand or be able to use the type-writer. She should be intelligent and neat in person and dress, and cannot be too big a crank in cleanliness to suit me. She should have a bright, cheerful disposition, as we have enough to ruffle us in a day's work, without having some one around who is sulky or morose. It helps wonderfully to have some one in the office of a sunny temperament. My assistant looks after the entire dental outfit, keeping everything, especially the instruments, absolutely clean, sees that instruments, etc., are always in the same place, so that there may be no time lost in looking around for anything when it is wanted. In a busy practice time is precious, and every moment must be conserved, besides our work moves along more smoothly when order prevails around the chair. I sometimes wonder how some men accomplish anything, when I see the topsy-turvy condition of their operating rooms. It is the assistant's duty to see that the stock is kept up, so that there may be no running to the supply house just when things are wanted, except it be for teeth. She attends to telephone calls, and sees all patients when they come in, and makes most of the appointments. She also keeps the records of operations and charges, and sends out the bills and receipts, and assists at the chair or bench in any way to facilitate the work and save time.

A professional man's time is largely his capital and this is especially so with the dentist. Having to apportion out our time, as we must for the different operations, the careful arrangement of the work to be taken care of each day is very important, and any measures we can employ to utilize our time in the most profitable manner should be taken advantage of. In fact, I know of nothing more important in the management of a dental practice than the intelligent and careful mapping-out and arrangement of the day's work. In a city practice, with one patient closely following another, two chairs are a great aid in utilization of time, even if they are both in the same room and only separated by a screen, though of course it is better to have the chairs in different rooms. While I have a regular hour for treatments, etc., which I shall speak of later, there are occasionally patients who require immediate attention, such cases I attend to in another room, shut off from the main operating room, and having a separate entrance from the reception room. This second operating room is very useful in many ways and I frequently leave a patient there after setting an inlay, for the cement to set, while I start work on the next patient in the other chair. I also use this second room when taking impressions, and instead of waiting while the assistant cleans up the plaster and makes things presentable, I can go right to work at the other chair. It is a good plan to have duplicates of those instruments that are in frequent use, such as mouth mirrors, explorers, scalers and some of the excavators. One set is always clean and ready, and when the soiled ones are removed may be brought in all ready for use. This saves much time from waiting, and averts the possibility of the assistant not properly cleansing them, as is not unlikely, when we are waiting for the instruments to go on with our work.

One very important item in the saving of time is the careful making out of a chart at the first examination of all the operations required, even if it be only one tooth, for it may be a week or more before the patient comes again, during which time many mouths have been worked on, and except in cases of wonderful memories it is not easy to remember the next time we see the patient which tooth or teeth required attention. There is a great deal of time lost in aimlessly looking around the mouth for the work to be done; sometimes the patient kindly points it out, but we should have our chart before us, then it can be seen at a glance what is required, provided, of course, that each operation is carefully checked off when completed. My assistant watches the appointment book, and has the patient's chart laid out before the chair is taken. All important in the management of practice is the care used in making appointments for operations, the arrangement of time for treatments and consultations, and avoiding interruptions. Engagements for operations should be made with the idea in mind of what we expect to accomplish at a given visit. In other words we should endeavor to map out the work in advance for each patient as fully as possible. I know it is not always possible when making an engagement to exactly time the contemplated operation, and perhaps it is better to be a little liberal in the allowance of time rather than err in the opposite direction; for if the operation does not quite take up the allotted time, there is frequently more in the same mouth to be done, or if not, the spare moments can generally be utilized in many ways before the next patient appears. In any case it is better than keeping a patient waiting too long, as we are apt to do, if sufficient time is not reserved for each operation. The more punctual we are in seeing our patients at the appointed time, the more punctual they will be in keeping their engagements. Then, too, while some men are not affected by the knowledge that a patient is waiting, there are some who are apt to hurry through with the work in hand and not make the operation as perfect as it should be. I might say in this connection that if you have one or a dozen patients waiting in your reception room, *forget them*, and concentrate your mind and energy only on what you are doing. Such a course will be a time-saver in the end, for your work will not then likely return a failure.

The day's work through will run more smoothly and more will be accomplished, if the appointments are so arranged that one patient follows another with little or no waiting. A half hour lost with one patient is hard to make up and frequently disarranges the day's program. So we must ask for punctuality from our patients, and encourage it by being as punctual ourselves as possible.

I speak strongly for method in our practice; it will make our work much easier and we will accomplish much more. Some men see patients at almost all hours for consultations and treatments, or will even leave the patient with the rubber-dam on to talk with an agent. There is no method in such practice, and patients get tired and sometimes indignant at such treatment and neglect. I think it best to set apart some hour of the day for consultations and treatments, say between twelve and one, or between four and five. It is understood by my patients that my hour for treatments, changing wedges, consultations, etc., is between twelve and one, and if they cannot come at that time, they generally come by appointment. I very seldom leave a patient I am operating for, except it be to give relief to one in pain. During long operations our patients may occasionally be glad to have us leave them for a few moments for a rest or slight relief, but as a rule, it is best to stick as closely to your operation as possible, without allowing interruptions to take you away; by so doing you will not disarrange your mapped-out time, and you will be apt to do better work. Concentration on what one is doing is very essential for success, and this is almost impossible if we keep leaving the chair.

An intelligent assistant will stand between us and unnecessary interruptions. I rarely leave my chair to see a patient who has not an appointment. In nearly all cases my assistant makes an appointment with them before they can see me. Regarding agents or others, who do not wish to see me professionally, they must send in their name or card so that I may know who they are, and what business they wish to see me on. If I am interested, an appointment is made at an hour which will not interfere with my work. If I am not interested they are told so, and are not wasting my time and their own by useless persistence.

At the first examination note carefully what is required to put the teeth in the best possible condition, considering the best means to bring about such a result, and after carefully mapping out the method and means to be employed, try and adhere to them as closely as possible. In other words, use method and don't go at any work slipshod, doing an operation here and there, not knowing what you are going to do next. Much more can be accomplished in a given time when we are systematic.

Regarding the breaking of engagements by patients without due notice, to my mindt, here is nothing more demoralizing in practice, and it is generally understood by my patients that failure to keep an appointment for operations, without timely notice, means that the lost time will have to be paid for. Such information is printed on my appointment cards, and it is closely lived up to. Of course, there are at times extenuating circumstances to be taken into consideration, and sound judgment must be used; but laxity in this direction is a mistake, and we will find as a rule that our patients value our time in about the same ratio as we do ourselves.

A few words now on the business side of our profession, viz, that of keeping records of operations, rendering bills, and collecting them; and I cannot refrain from saying here that many of us make a very poor business of it indeed. If do we not see that we are paid for our services, and that, too, with some degree of regularity, how can we hope to be successful? If we are not paid for our work, how can we pay our own bills, and keep up our good standing in the community in which we live? A dentist who pays his bills promptly, and does not have debts continually hanging over his head, is in a much healthier state of mind generally, and much better fitted to cope with the daily difficulties of life.

The simpler our method of bookkeeping, the better. My accounts and records of operations are kept by the card system by my assistant. I keep a day-book or journal in which is entered the day's work, credits and debits, from which book the ledger card system is made up. By using the card system we avoid the handling of a bulky ledger and also avoid having to look through perhaps several hundred pages monthly to find the unpaid accounts. There are three sets of cards, each of different color, alphabetically arranged, one color for unfinished operations, one for finished operations which have not been paid for, and one for paid-up accounts. All that is necessary at the end of the month is to look through one set of cards to get a full list of those whose work is finished but for which payment has not been made. Bills are mailed once a month. That is, a bill is sent the first of the month following that in which the work has been completed, and payment is requested by the fifteenth. If no notice is taken of the sending of a second or third statement, I call attention to it and ask for payment, though we must use discretion in this as in anything else. I can see no reason for allowing an account to run along indefinitely before rendering a bill, nor can I see any reason why reasonably prompt payment should not be requested after the bill is rendered. If we are lax in our methods of sending out statements, and seeing that they are paid within a reasonable time, we will soon find our patients just as lax on their part. Then, too, when a bill is rendered promptly, the extent of the work with its difficulties, etc., is fresh in the patient's mind, frequently accompanied by gratitude for the service rendered, and payment is promptly and willingly made, whereas, if several months elapse before the bill is sent, only a dim recollection remains of what has been done, and the bill at that time may look large. Remember, old bills are always the hardest to pay and to collect. I do not render any itemized statements, unless requested to do so, which is very rarely the case. The statements just read "For professional services from such a date to such a date." The patient is not much wiser as a rule for receiving an itemized statement, and to me it takes on somewhat of a commercial transaction, rather than a professional one as it should.

I believe I have fully reached my time limit, though this subject, if treated thoroughly in all its phases, is one that would furnish ample material for a much longer paper than this.

I can hardly close without again referring to the need of being punctual in all our office affairs, not forgetting that good business methods are not to be despised because we are professional men. We must not forget that the building up of a good practice and retaining it is only accomplished by faithful, conscientious work, and close and careful attention to the many little things that make for successful office management.

### DISCUSSION.

Dr. Hall: The paper is so complete that I can add little. I would like to emphasize the suggestion of sending out statements regularly and promptly. I have often had trouble in collecting fees when I have delayed rendering the bill so long that the patient has forgotten the character of the service and thought an over-charge had been made. Whereas, had I sent the bill while his memory was clear on the matter he would not have demurred or questioned my bill. It is a good plan also to make a memorandum on the ledger every time a statement is sent out, and to inform the patient to whom several bills have to be sent of the dates when former bills which are still unpaid were sent him. It begets confidence as to accuracy, and is liable to induce the patient to do likewise.

Dr. Loeffler: Some dentists seem to think it is a good plan to have the office always full of waiting patients. I never could feel that I had any right to use up my patients' time in this way. Of course, times will occur that patients are unavoidably kept waiting, but patients should not be encouraged to think they can come in and interrupt you when you have agreed to give your time to some one else, whose time may be valuable to them. It is also bad practice to keep patients who are suffering, waiting while you gossip or delay with matters which may easily be postponed. A little thoughtfulness and tact will enable one to keep the office clear and the work running smoothly and continuously.

DR. LATHROP: I do not hesitate to send statements to my patients at the first of the month or any other time. It may seem like commercialism, but there is no reason why it should not be done. I also do not hesitate to render statements at the beginning of the month, even if the work is still incomplete. The character and bearing of the lady office assistant is a very important matter. She stands between the operator and the public and saves him a lot of annoying visits, and, if she is tactful and knows her business, will often save her wages in a day. I think these girls are very often repulsive or at least objectionable because of their personal appearances. They frequently are either over-dressed or untidy. One is about as objectionable as the other. I have seen a girl in a dental office dressed as though she were going to a party. This is as bad taste as though she were untidily dressed. Appropriately dressed and of affable disposition, they can be of great help.

DR. WOOD: I am accustomed to sending my patients an itemized statement of account, and never knew of one objecting to it. I even go further and enclose a marked chart. I send my statements when the work is completed, whether it is in the middle of the month or the first day.

DR. HILDRETH: We get our bills the first of each month from business houses and it has become so customary that it seems a proper time to render bills. It is the time when salaries and wages are paid, and most people would like their bills at that time, so they can pay them with the least bother. It is a good plan to send all bills at that time.

DR. SPRING: I have a method of inducing patients to pay at once. As each operation is completed, I mark it on a chart and ask them to hand it to the assistant, who is seated at the desk and prepared to take payment. The patients will either pay or arrange a time for the payment. This saves bookkeeping and makes collections more prompt.

DR. HOFF: The character of an assistant is vastly more important than her dress. As Dr. Lathrop has said, she must stand between the dentist and the public. In order that she may do this she should know the business thoroughly and also how to meet and dispose of people who come to the office in the best possible manner. She must have a high order of intelligence and be gracious and tactful and have a deep interest in the affairs of the office. Such an assistant will be able to make appointments, receive and dismiss patients, or entertain them while waiting. She can also keep books and collect fees and any other routine business. With proper training, she can be very useful at the chair, keeping instruments and other appointments in order, and ready for instant use. There is no reason why she may not keep cutting instruments sharp and ready for use. It is not impossible to have an assistant who can perform some of the simpler operative procedures. It is not to be expected that an assistant, who can do all these things well, can be employed at a small salary. They should command good pay and be thus encouraged to increase their usefulness. The more intelligent, the more profitable they will be.

